# Construction of a rural tourism information service management system for multi-source heterogeneous data processing

**DOI:** 10.7717/peerj-cs.1334

**Published:** 2023-06-09

**Authors:** Xuefei Wu, Jiahui Huang

**Affiliations:** 1 Academic Affairs Office, Tourism College of Zhejiang, Hangzhou, Zhejiang, China; 2 Zhejiang Academy of Culture & Tourism Development, Tourism College of Zhejiang, Hangzhou, Zhejiang, China

**Keywords:** Rural tourism, Data storage, Information management, Netty

## Abstract

This study offers an integrated service management system for rural tourist information based on a cloud platform to address the three main issues of high platform concurrency, difficulty storing and managing data, and trouble sharing data functions. Three levels—data, process, and architecture—are considered in the analysis and design of the platform. The Hadoop data storage system makes possible the collection, storage, administration, and exchange of data functions for large amounts of heterogeneous data from many different sources by utilising Netty data transmission technology, hybrid data storage technology, and the Web Foundation. The results demonstrate that the system’s response time is low, and the CPU consumption time and the average utilisation rate meet the actual needs. They resolve issues with the current rural tourism platforms application, such as the difficulty of data collection, the low rate of reuse, the low rate of sharing, the lack of timely updates, and severe island phenomena.

## Introduction

The state has attached great importance to tourism development in various regions in recent years and constantly introduces new development measures. As a part of intelligent tourism, characteristic rural tourism has become essential to supporting the economy (Shiyun, Xingyan & Zhenxing, 2016; Wen, 2016). Managers use a method of driving the overall situation at the regional level and a specific driving economy to try to create an intelligent tourism industry chain to improve tourism. The managers also actively seek development ideas and launch township- and even village-level characteristic tourism projects using each county as the unit. They also continuously introduce typical rural tourism, characteristic tourism, and rural culture projects. But at present, rural tourism development still needs to be improved. Rural tourism resources are scattered, and most data are from isolated islands. Although some scholars can collect data, they do not have the idea of serving the public, so it is still difficult for users to obtain rural tourism information (Qian, Huanhuan & Xixi, 2021; Ming & Xuelei, 2021). Therefore, the structure of intelligent tourism with the help of the Internet meets the requirements of the current economic development of various regions. The cloud-based platform has become the core of the development of tourism.

Some experts put forward more suggestions for the research of tourism management systems. Through comprehensive analysis of relevant research results worldwide, they put forward that smart tourism should be guaranteed by appropriate technologies to provide practical help for relevant tourism information, tourism routes and various reservation services. It is considered that the government and relevant tourism management departments should provide appropriate assistance as the starting point, and do an excellent job in transportation service providers, tourism commodity providers and other service providers (Li et al., 2017; Ruifeng, 2013). The functions involved in the system include acquiring tourist weather, intelligent navigation, and a product shopping guide, which lets tourists genuinely experience the convenience, speed and service quality brought by smart tourism. Although the development direction of China’s tourism has been established, it does not have the regional characteristics and has not formed the critical management and service of rural tourism but focuses more on publicity. There has been a lot of research on big data in rural tourism (Jin Jin & Wei Wei Wei, 2019; Wenlin & Min, 2015; Ying, Zhi & Juan, 2014), but there is no centralized for updating the data. The majority of data gathered by the government, companies and researchers are private. There is a lack of a mechanism for data sharing. The visual aids are not discretely and methodically grouped; on the big data cloud platform for rural tourism, there are no unified standards or comprehensive data. Therefore, this article uses cloud platform technology to solve the bottlenecks. The main contributions of this article are as follows: (1) A rural tourism information management system based on a cloud platform is proposed based on in-depth research and rural tourism development research. (2) Through software and hardware design, it realizes collection, storage, management and data sharing of massive multi-source heterogeneous data.

## Key Technologies

### Data transmission based on Netty

With the continuous gathering of tourism platform data, the rural tourism collection subsystem will collect more and more data, and the data transmission within the platform will be more and more frequent. Suppose the traditional data transmission mode is used for data transmission. In that case, the server will switch threads frequently due to too many opened lines, which will cause many server resources to be occupied (Gao et al, 2018). As a result, all systems of the rural tourism cloud platforms are facing paralysis. At the same time, data reading and writing efficiency are low in the blocking I/O (BIO) network mode. This is because the data reading and writing is stream-oriented and can only read and write data character by character, which makes the efficiency slow. In this way, the bio network mode is no longer applicable in the case of frequency response with the server.

**Figure 1:**
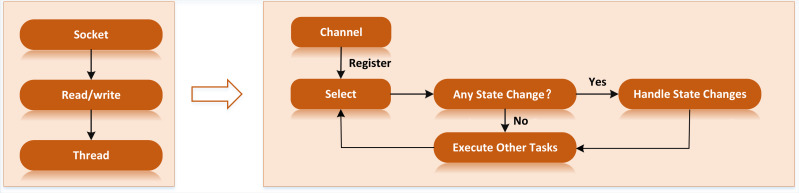
NIO information processing flow. Once a socket is established, it will be submitted to the thread, waiting for the line to process it and then return it to the user (Campello et al., 2015).

Therefore, the platform has modified the data transmission mode, using the non-blocking I/O (NIO) method to complete the data transmission. NIO has three core components: buffer, selector and channel. The buffer is a memory buffer, the channel is an operation channel to access I/O, and the selector is a multiplexer to manage multiple channels by one thread (Guangfu, 2021). In NIO, the line does not accept sockets directly. Instead, the request will be sent to the selector, and the selector will traverse all the sockets. Once a socket is established, it will be submitted to the thread, waiting for the line to process it and then return it to the user (Campello et al., 2015). The NIO information processing flow is shown in Fig. 1 below.

The rural tourism data transmission technology based on Netty can avoid the system crash caused by the super high concurrency of the server, which is essential for the operation of the rural tourism cloud platform.

### Data storage technology

The rural tourism cloud platform designed in this article adopts the storage mode of MongoDB NoSQL database and Hadoop distributed file system based on distributed data storage tools. Its system is composed of multiple computer nodes, which can communicate and coordinate with each other.

#### MongoDB database

**Figure 2:**
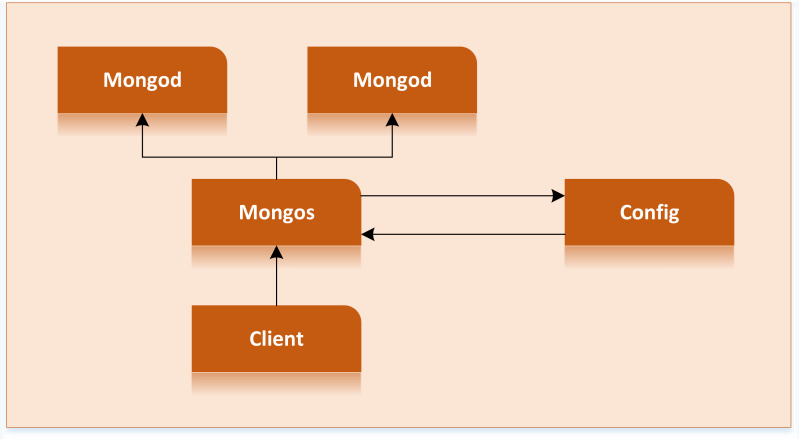
Cluster structure of sharding. When the chunk exceeds the set size, the background will automatically segment the piece to avoid the system crash caused by a too-large piece.

Since MongoDB is document oriented and supports data storage with a disordered structure, it fits well with the data characteristics of rural tourism, which is very suitable for storing rural tourism data (Nicolau, 2021). At the same time, it has a sharing method, which can disperse rural tourism data to other computer servers and is more advantageous for large data sets and high loads. Even if the performance of each server is poor, it can support the rapid growth of data volume and load by increasing the number of servers. MongoDB automatically divides the data into chunks, where each chip represents part of the information on rural tourism. When the chunk exceeds the set size, the background will automatically segment the piece to avoid the system crash caused by a too-large piece. Figure 2 shows the structure of sharding clusters (Abu Kausar, Nasar & Soosaimanickam, 2022).

#### Hadoop system

In addition to MongoDB distributed storage tools, Hadoop distributed file system is also used to construct data storage subsystem (Merceedi & Sabry, 2021). This is because with the operation of the rural tourism cloud platform, various types of data in the forum will become larger and larger as time goes on, and the efficiency of data retrieval by MongoDB NoSQL database will be lower and lower. Moreover, due to the lack of regular collection of rural tourism data scheduling, it is easy to make the subsequent development of data query and analysis functions lack solid support. As mentioned above, to avoid problems in the rural tourism platform, the Hadoop system provides storage, management, query and analysis services for these data.

**Figure 3:**
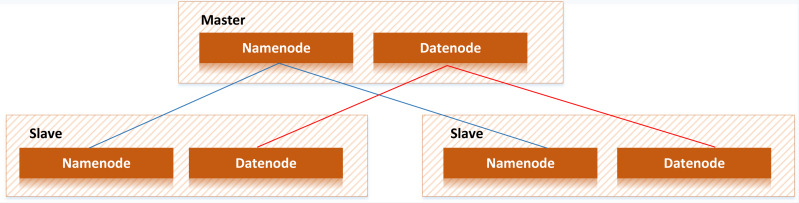
Hadoop structure. Hadoop is mainly composed of HDFS and MapReduce. HDFS adopts master/slave architecture. There is one Namenode node and multiple Datanode nodes in the HDFS cluster, while Namenode is used to manage Datanode, and Datanode is used to store actual data (Merceedi & Sabry, 2021).

As shown in Fig. 3, Hadoop mainly comprises HDFS and MapReduce. HDFS adopts master/slave architecture. There is one Namenode node and multiple Datanode nodes in the HDFS cluster, while Namenode is used to manage Datanode, and datanode is used to store actual data (Lin, Chen & Luo, 2021).

HDFS is the underlying foundation of Hadoop to store massive rural tourism data. HDFS sliced the rural tourism historical data into chunks and kept them in different rural tourism servers (Alange & Mathur, 2019).

### Web framework

**Figure 4:**
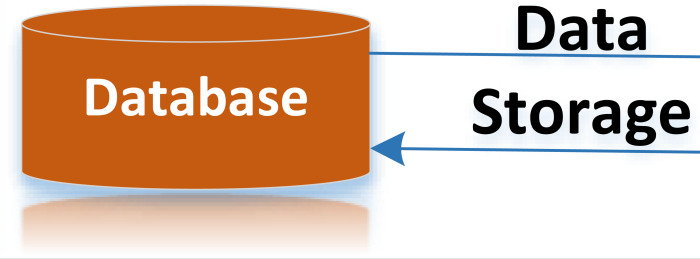
Workflow of web framework. Django’s workflow is that first, use the manage. py runserver command to start the Django server and load setting.py starts the corresponding access port at the same time.

We require a tool that can rapidly and effectively interface with the front-end rural tourism service platform subsystem and the background rural tourism data storage subsystem to construct a cloud platform for rural tourism. At the same time, Django may be more effectively connected with the finished part because Django is primarily used for programming design in rural tourist platforms (Liawatimena et al., 2018; Gore et al., 2021). The Model-View-Template (MTV) mode is Django’s software development foundation. Figure 4 illustrates the Django procedure. To start the Django server and load settings, use the manage.py runserver command, py simultaneously opens the matching access port. Django will check the visited port against the current port in the background after the user views the associated interface and then call the pertinent function to execute after success; After the process is finished, the data can be sent to the browser as an HTTP Response so that users can explore and launch the entire cloud platform for rural tourism.

## Design of Rural Tourism Information Management System

There are three key technologies: rural tourism data transmission method based on Netty, mixed rural tourism data storage technology and rural tourism web framework. The application of these key technologies effectively solves the problems of difficult storage and scheduling of massive data encountered in the construction of rural tourism cloud platforms, realizes the rapid interaction and rendering of data between the subsystem of data storage and the subsystem of service platform, and lays a technical foundation for the development and use of data sharing mechanism.

### Architecture design

#### Overall architecture

**Figure 5:**
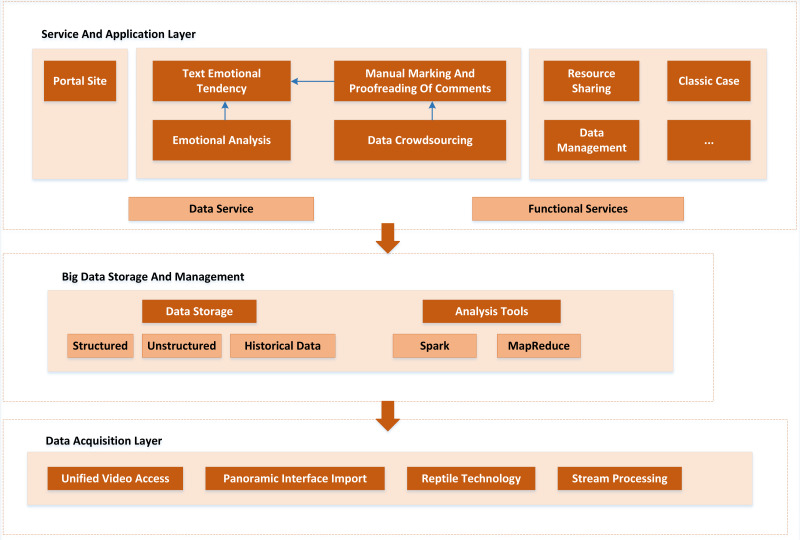
Overall architecture. The overall architecture of the cloud platform is divided into four parts: the data acquisition layer, the big data storage and management layer, the service and application layer, and the security system.

Because there are many modules on this platform, it is difficult to maintain the code to avoid the confusion of platform construction. Therefore, before the construction of the platform, the overall design of the platform architecture is made. The overall architecture of the platform is divided into four parts: the data acquisition layer, the big data storage and management layer, the service and application layer, and the security system. The overall architecture of the cloud platform is shown in Fig. 5.

As a continuous provider of multi-source heterogeneous cloud platform data, the data acquisition layer mainly includes basic scenic spot information, video audio, panoramic photos, microblog comments, Ctrip comments, and rural tourism-related news and activities information in the official websites of prefecture-level cities and scenic spots. The data collection is mainly realized by Python-based web crawler technology. At the same time, some data need to be collected manually or generated automatically by the platform, such as basic information about scenic spots, system logs, *etc*.

The primary objective of big data storage and management is to achieve unified storage, mutual scheduling and effective management of rural tourism. It provides adequate data support for the cloud platform to build a harmonious and standardized sharing mechanism. At the same time, according to the different characteristics of multi-source heterogeneous rural tourism big data, appropriate storage methods should be used for storage, and a unified rural tourism visualization management and query demonstration application should be established.

All platform services are supported by the service and application layers. Additionally, when utilizing the platform, users can directly access these tiers. The following services make up the platform’s main business units: By getting access to the service layer’s API service—the phrase used to describe how the platform developers package the data and resources in the platform into API services—the application layer can indirectly access the rural tourism data storage subsystem. Security assurance system mainly refers to avoiding the frequent access of external users to the open and shared API interface, resulting in excessive pressure on the system server and affecting the system’s regular operation (Nandigramwar et al., 2021; Le & Pishva, 2015).

#### Data architecture

Data is the starting point and core of the platform construction. The data sources of the platform are summarized into five categories: basic information about scenic spots, data on scenic spot reviews, media information about scenic spots, virtual reality information about scenic spots and user behavior data. Due to the different characteristics of the five data types, storing the data without resolution will lead to data confusion, difficulty in management and data redundancy. To solve the above problems, the five data types are summarized and divided into scenic spot, behavior, and platform data. The rural tourism data storage subsystem is established to unify planning data collection and storage methods. According to the actual data characteristics, different data collection methods and storage methods are adopted, and unified standards are formulated, which realize the efficient storage and management of data. When the rural tourism data acquisition subsystem works, it also needs to conduct data docking with the data storage subsystem in addition to using its own multiple collectors. In this process, the collected data is uniformly connected to the database through communication services.

Afterward, through the construction of the rural tourism service platform subsystem, it provides dynamic analysis, word cloud analysis and other functional interfaces and data resource sharing interfaces to realize the exchange and sharing of data and functions. It can enhance the sharing ability of data and operations of the cloud platform and facilitate the client to call data and function resources more conveniently.

#### Application architecture

**Figure 6:**
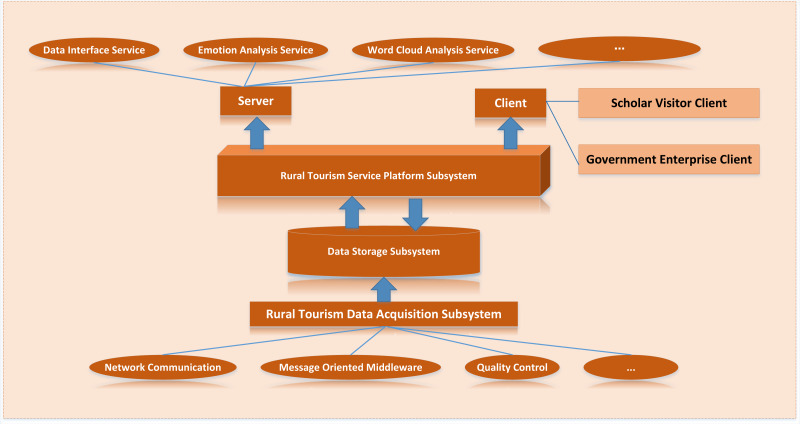
Application architecture design. The application architecture of the platform is shown, which can be divided into rural tourism data acquisition subsystem, rural tourism data storage subsystem and rural tourism service platform subsystem.

According to the overall architecture design, the rural tourism cloud platform consists of a data collection layer, big data storage and management layer, service and application layer and security system. However, to complete the actual landing development of the platform, it is necessary to build corresponding subsystems to realize the business functions. The application architecture of the platform is shown in Fig. 6 below, which can be divided into rural tourism data acquisition subsystem, rural tourism data storage subsystem and rural tourism service platform subsystem.

The rural tourism service platform subsystem can be divided into server and client. The server is responsible for transforming the data of the data storage subsystem and the functions developed in the platform into accessible service API through the Django framework for internal application and sharing with external users. The client includes a government enterprise client and a scholar visitor client. According to the different functional requirements of the two kinds of clients, the corresponding API in the server is read respectively, and the client website of the service platform subsystem is finally formed by rendering the web page.

### Functional design

#### Data acquisition subsystem

The rural tourism data acquisition subsystem must be connected to the data storage subsystem to use its many collectors. The obtained data are combined into the database using the communication service in this step. Because these network data are generated at different rates daily, the data acquisition terminal must select different collection periods based on different speeds, such as the slower generation rate of Ctrip and news and the faster generation rate of microblog and environmental data. The actual data update for this article can be found in Table 1, and it provides a general outline of the collection cycle of several data types.

#### Data storage subsystem

The rural tourism data storage subsystem is the transition between the data acquisition and service and application layers, which can be divided into the storage and quality control parts.

The quality control part is an essential cornerstone of rural tourism data storage to ensure the correctness and reliability of the data. Due to the complexity of the data on the Internet, reliability is low without quality control. Therefore, a quality control module is developed for microblogs, Ctrip and news, which can automatically monitor and control the reliability of the access platform data from the server back-end to ensure the accuracy and reliability of the data in the platform.

**Table 1:** Data acquisition period. In this article, the actual data update has preliminarily drawn up the acquisition cycle of various data types.

Data type	Micro-blog	Ctrip	Environmental data	Media data
Cycle/day	7	30	1	30

#### service platform subsystem

Both the server and client have a data storage subsystem to provide data support, and the Django framework is used to build the server within the platform. At the same time, the client also needs to use The Django web framework to interact with the back end. The server mainly provides data and available services for the platform client and establishes a unified and standardized data-sharing mechanism (Burch, 2010).

The client provides corresponding functions for different user types. Users can provide services directly through the platform, dynamic analysis, and word cloud analysis. After the model training is implemented through the service API, the test set calculation results are input and uniformly calculated by the cloud platform. Users can directly obtain the result data after completion.

The server includes two aspects: data service and function service. The function service mainly includes emotion analysis service and word cloud analysis service. Affective analysis service provides two sentiment analysis methods: text sentiment orientation discrimination based on syntactic rules and sentiment dictionary and text sentiment recognition based on deep learning (Kumar & Raman, 2022). Users can also adjust the relevant parameters through the visual interface to improve the effect of text emotion recognition or directly conduct dynamic analysis according to the default parameters provided by the system to get the comprehensive emotional tendency in the scenic spot. Word cloud analysis mainly uses the MapReduce tool of Hadoop to realize the rapid statistics of word frequency of rural tourism text to quickly analyze the comment hot spots of a specific time or region.

Government enterprises and student travelers are two different groups in the market. The government enterprise side has developed function modules. These include collection management, data management, emotional map, dynamic analysis, open approval, *etc*., to help the relevant users of government enterprises fully manage the picturesque areas for rural tourism within their control. The scholar tourist terminal built the home page, personal center, tourism map, resource center, data crowdsourcing, and other helpful modules. Through the analysis and study conducted utilizing the platform’s data and function ports, which are available to the public, users can gain more knowledge about the general status of rural tourism.

## Realization of Subsystems

### Data acquisition subsystem

First, the website of rural tourist attractions on Ctrip was collected manually, and a total of rural tourist attractions were collected on the official website of Ctrip https://www.ctrip.com/) 156 addresses. Then, use selenium 3141 + webriver + chromedriver to simulate the scene of users using the Ctrip tourism platform. Through automatic access, click and slide, collect the website source code and analyze and sort out the required data. Because of the massive amount of code, only part of the background code of the Ctrip collector is shown for reference, as follows:

**Table:** 

Algorithm 1
#Collector start module
try:
Print (“implement [Ctrip review] crawling)”
xiech_comment_get_mian()
item = str(now) + &apos;—XIECH—SUCCESS—&apos; + &apos;&apos; + &apos
;\n&apos;
writeLog(&apos;spiderLog.txt&apos;, item)
except Exception as e:
print(e)
item = str(now) + &apos;—XIECH—ERROR—&apos; + str(e) + &apos;\n&apos;
writeLog(&apos;spiderLog.txt&apos;, item)
#Analysis and arrangement module
product = {
‘user ID’: Data_Resize.name_get(user_info),
‘score ’: Total_score,
Detail score: small_score,
Data’ comments’_Resize.commentDetail_Get (user info), # comment details
‘picture’: Data_Resize.picture_get(user_info),
‘publish time’: Data_Resize.publish_time_Get (user info), # release time
}

### Data storage subsystem

The data storage module of the rural tourism data storage subsystem mainly uses MongoDB. It is suitable for storing user information, environmental parameters and other information in scenic spots, scenic spot basic information, microblog data, Ctrip data, news information, system logs and other data with time sequence and high frequency. Taking the environmental parameters as an example, it is appropriate to use the MongoDB database to store such long-time series data because of its fast updating, high frequency, and real-time data characteristics. The data file structure of regional weather and air quality information is as follows:

**Table:** 

{
_ID: scenic spot ID,
Name: the name of the scenic spot,
Start_Date: the date of the earliest collection,
End_Date: the date of the latest collection,
Data: [{time ’: ’time of the day, ‘weather ’: ‘acquired value’,’ real-time temperature ’:’ obtained
“Wind direction”, “wind speed”, “acquired value”, “humidity”, “acquired value”,
“Visibility”: acquired value “,” air pressure “,” acquired value “,” air quality “,” acquired value ”,
“Air quality value” obtained
}

### Service platform subsystem

The specific workflow of the server interface is as follows: the system background service is developed by the DjangoMVT framework, the rural tourism data storage subsystem provides data support for service API, the front-end sends HTTP requests to the server through browser Ajax, which is the API interface to call back-end services (for example, https://zenodo.org/record/5643728#.ZEYYjXZBzGI; when the server receives the request, it maps the corresponding method show according to the path “index / GL / Weibo.” After confirming that the operation is correct, the query can be processed, and then the results are packaged into JSON format data and returned to the front end. Finally, the front end gets the data for rendering and presents it to the user.

Government agencies and student tourists make up the majority of the clientele. The front-end framework of the enterprise government client and the scholar tourist client is built using front-end languages such as HTML, CSS, and JavaScript with the assistance of the server side of the data storage subsystem and the service platform subsystem. The client functions are examined and created based on the functional requirements. The built-in front-end client is then integrated with the back-end server using Django, and clients for rural tourism government enterprises and rural tourism scholar tourists are achieved.

## System test

### Development environment

In terms of server configuration hardware in the development process, the network bandwidth uses 100 m shared bandwidth, and the server CPU uses Zhiqiang v5608, 3.5 GHz, four core. The memory is DDR4 16g memory, hard disk 320G capacity. The number of servers is four, which are used to manage applications, files, databases and videos.4G memory is used in the computer configuration of the development side, Pentium G5680 3.5 GHz, hard disk 120 g solid state disk. After the development of the system, it is released to the server for testing.

### Performance test

**Figure 7:**
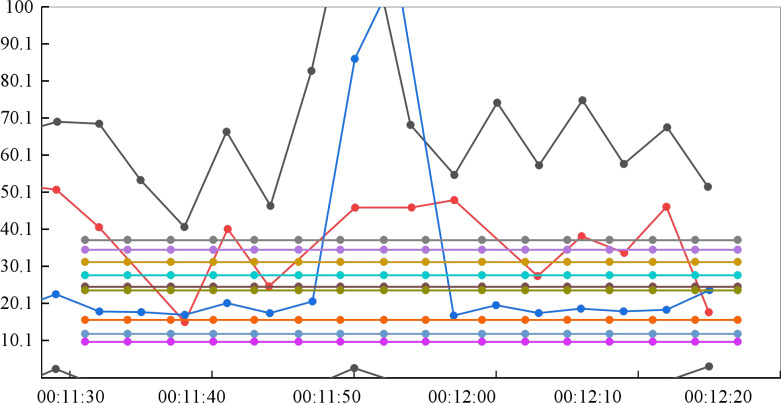
Test results of server resource occupancy. The interval duration is set to 10 m for the particular test connection, which means that 20 concurrent users will be added automatically every 10 min to track changes in system performance characteristics.

The system’s concurrent access does not imply that it will allow access from many users simultaneously, but that new users will continuously join. The interval duration is set to 10m for the particular test connection, which means that 20 concurrent users will be added automatically every 10 min to track changes in system performance characteristics. Figure 7 displays the server occupancy test results.

The maximum CPU usage is 13.189%, which is lower than the average of 80%. There is no hardware bottleneck in the system’s virtual environment, as indicated by the hard disk’s maximum and minimum values of 0.332 and 0.000, respectively, which fall within the usual range of 00 to 20.

Every 10 s, five concurrent access users are added to the test script so all simultaneous users can simultaneously utilize a specific platform function. As more concurrent users are added, the performance metrics of the application server are tracked. To ensure that the system can scale, 500 simultaneous access users are tested without considering time while measuring the system’s performance. The actual number of concurrent users on the platform will be 300.

The system’s minimum response time of 5S and average access time of less than 2.959s, respectively, fulfill the actual needs. While the average calculation is less than 12.626%, which follows the usual requirements, the CPU utilization rate of the system reaches 57.553% when concurrent users request many data. The findings indicate that the total contemporary access process’s average memory consumption rate is 680m, which is within the acceptable range and satisfies the actual needs.

The system contains 130 test cases in total. In the end, 130 test cases are conducted after extensive testing, of which 102 are successful and 28 are not done correctly. After examination, it has been determined that several of the 28 test cases were unsuccessful because of flawed system function flow designs, others were due to incorrect system page links, and a few were due to data format inspection without judgment.

The system performance test primarily examines throughput, CPU time used for database operations, CPU utilization, average transaction response time, and other metrics. Table 2 displays the performance test results after data from the whole testing process were analyzed and statistics were calculated.

**Table 2:** Results of the performance test. The table displays the performance test results after data from the whole testing process were analyzed, and statistics were calculated.

Test items	Result
Average transaction response time	64.834s
Number of transactions processed by the system per second	60
CPU consumption time	70.93%
Average CPU utilization	15.625%
Remaining available memory	30%
Network throughput	685

The results show that the system’s response time is low, and the CPU consumption and average utilization can meet the actual needs.

The system adopts the SSM framework, which is convenient for the classification and management of data information and reduces the amount of code to write programs to a large extent. Then the text emotion algorithm is combined with the scenic spot recommendation system, which can achieve the synergistic effect in scenic spot recommendation and applies to the travel recommendations. With the increase in historical behavior of user comments, the synergistic effect will be gradually obvious, to better meet the individual needs of users and, to a large extent, improve the efficiency of information retrieval.

## Conclusion

This study develops a comprehensive service management system for cloud-based information on rural tourism. The data storage subsystem solves the problems of rural tourism data in multi-source heterogeneous data storage, transmission, and management and realizes the unified storage and effective management of rural tourism data by using MongoDB and Hadoop hybrid data storage mode and using the Netty framework to build an efficient transmission server. The service platform subsystem can assist government enterprise users with the efficient management of scenic areas and platform data, as well as with the rapid acquisition of rural information for researchers and the provision of all-inclusive rural tourism services for visitors. The test results demonstrate that the system has a fast response time and that its average CPU utilization and time may match the actual demands. However, the method proposed in this article does not perform a horizontal comparison of various scenic locations in a given area, which is also the future research focus for system optimization. Due to anti-crawler means on some websites, some data types cannot be read directly by the interface and can only be collected by selenium + webdriver and other means. While the Internet update iteration speed is fast, the code needs periodic maintenance to avoid the failure of the crawler and the failure of normal data collection.

##  Supplemental Information

10.7717/peerj-cs.1334/supp-1 Supplemental Information 1 Raw data

10.7717/peerj-cs.1334/supp-2 Supplemental Information 2 Code

## Additional Information and Declarations

### Data Availability

The following information was supplied regarding data availability: The code and raw data are available in the Supplementary Files.
