# Supplementary material for: Construction of a rural tourism information service management system for multi-source heterogeneous data processing
Source: PeerJ Comput Sci. 2023 Jun 9;9:e1334. doi: 10.7717/peerj-cs.1334 (PMC10280680; doi:10.7717/peerj-cs.1334)
Supplement: Supplemental Information 2 [file peerj-cs-09-1334-s002.zip › Code/webmagic-core/src/main/java/us/codecraft/webmagic/selector/package.html]

Selectors for page extraction. Core API is the interface Selectable，and internal core is the interface Selector。
