# Supplementary material for: Construction of a rural tourism information service management system for multi-source heterogeneous data processing
Source: PeerJ Comput Sci. 2023 Jun 9;9:e1334. doi: 10.7717/peerj-cs.1334 (PMC10280680; doi:10.7717/peerj-cs.1334)
Supplement: Supplemental Information 2 [file peerj-cs-09-1334-s002.zip › Code/webmagic-core/src/test/resources/html/mock-github.html]

code4craft/webmagic


Skip to content

This repository

- Pull requests
- Issues
- Gist

- New repository

  New organization

  This repository

  New issue

  New collaborator
- Signed in as **code4craft**

  Your profile

  Your stars

  Explore

  Integrations

  Help
  Settings

  Sign out

- Unwatch

  367

  Notifications

  Not watching
  Be notified when participating or @mentioned.


  Watch

  Watching
  Be notified of all conversations.


  Unwatch

  Ignoring
  Never be notified.


  Stop ignoring
- Unstar

  1,743


  Star

  1,743
- Fork

  ## Where should we fork this repository?

  1,128

# code4craft/**webmagic**

Code


Issues
67


Pull requests
14


Wiki


Pulse


Graphs


Settings

A scalable web crawler framework.
http://webmagic.io/
— Edit


Description

Website

Save
or Cancel

- 698
  commits
- 6
  branches
- 13
  releases
- 23
  contributors

1. Java
   72.2%
2. CSS
   11.6%
3. JavaScript
   8.5%
4. FreeMarker
   7.4%
5. HTML
   0.2%
6. Ruby
   0.1%

Java
CSS
JavaScript
FreeMarker
HTML
Ruby

New file

Find file

SSH

Choose a clone URL

HTTPS
(recommended)

Clone with Git or checkout with SVN using the repository's web address.


HTTPS

SSH

Clone with an SSH key and passphrase from your GitHub settings.


SSH

Learn more about clone URLs

Download ZIP

*Branch:*
master

Switch branches/tags

- Branches
- Tags

0.4.x


0.6.0


en-webmagic


gh-pages


master


stable

Create branch: 
from ‘master’

webmaigc-0.4.3


webmagic-parent-0.3.1


webmagic-parent-0.2.1


webmagic-0.4.2


webmagic-0.4.1


webmagic-0.4.0


webmagic-0.3.2


webmagic-0.3.0


version-0.2.0


version-0.1.0


WebMagic-0.5.2


WebMagic-0.5.1


WebMagic-0.5.0

Nothing to show

New pull request

Latest commit
800f66c
Jan 18, 2016


code4craft
Revert "remove some unkown config"

…


```
This reverts commit 0e245c9.
```

Permalink

|  |  |  |  |
| --- | --- | --- | --- |
|  | Failed to load latest commit information. | | |
|  | assets | 同步官方源码 | Apr 12, 2014 |
|  | en\_docs | docs | May 3, 2014 |
|  | webmagic-avalon | update version to snapshot | May 5, 2014 |
|  | webmagic-core | 修正FileCacheQueueScheduler导致程序不能正常结束和未关闭流 | Nov 12, 2015 |
|  | webmagic-extension | Merge pull request #237 from SpenceZhou/master | Dec 2, 2015 |
|  | webmagic-samples | Merge pull request #227 from hsqlu/master | Jan 16, 2016 |
|  | webmagic-saxon | update version | Jun 4, 2014 |
|  | webmagic-scripts | update version | Jun 4, 2014 |
|  | webmagic-selenium | update and validate pom.xml | Jul 11, 2015 |
|  | zh\_docs | contributor | Jun 4, 2014 |
|  | .gitignore | change\_gitignore | May 19, 2014 |
|  | .travis.yml | remove ci for jdk6 | Jan 18, 2016 |
|  | README.md | contributor | Jun 4, 2014 |
|  | pom.xml | Revert "remove some unkown config" | Jan 18, 2016 |
|  | release-note.md | #34 Close reader in FileCacheQueueScheduler | Nov 8, 2013 |
|  | user-manual.md | deperate in user manual | May 3, 2014 |
|  | webmagic-avalon.md | scripts readme | Nov 28, 2013 |

### README.md

Readme in Chinese

User Manual (Chinese)

> A scalable crawler framework. It covers the whole lifecycle of crawler: downloading, url management, content extraction and persistent. It can simplify the development of a specific crawler.

## Features:

- Simple core with high flexibility.
- Simple API for html extracting.
- Annotation with POJO to customize a crawler, no configuration.
- Multi-thread and Distribution support.
- Easy to be integrated.

## Install:

Add dependencies to your pom.xml:

```
<dependency>
    <groupId>us.codecraft</groupId>
    <artifactId>webmagic-core</artifactId>
    <version>0.5.2</version>
</dependency>
<dependency>
    <groupId>us.codecraft</groupId>
    <artifactId>webmagic-extension</artifactId>
    <version>0.5.2</version>
</dependency>
```

WebMagic use slf4j with slf4j-log4j12 implementation. If you customized your slf4j implementation, please exclude slf4j-log4j12.

```
<exclusions>
    <exclusion>
        <groupId>org.slf4j</groupId>
        <artifactId>slf4j-log4j12</artifactId>
    </exclusion>
</exclusions>
```

## Get Started:

### First crawler:

Write a class implements PageProcessor. For example, I wrote a crawler of github repository infomation.

```
public class GithubRepoPageProcessor implements PageProcessor {

    private Site site = Site.me().setRetryTimes(3).setSleepTime(1000);

    @Override
    public void process(Page page) {
        page.addTargetRequests(page.getHtml().links().regex("(https://github\\.com/\\w+/\\w+)").all());
        page.putField("author", page.getUrl().regex("https://github\\.com/(\\w+)/.*").toString());
        page.putField("name", page.getHtml().xpath("//h1[@class='entry-title public']/strong/a/text()").toString());
        if (page.getResultItems().get("name")==null){
            //skip this page
            page.setSkip(true);
        }
        page.putField("readme", page.getHtml().xpath("//div[@id='readme']/tidyText()"));
    }

    @Override
    public Site getSite() {
        return site;
    }

    public static void main(String[] args) {
        Spider.create(new GithubRepoPageProcessor()).addUrl("https://github.com/code4craft").thread(5).run();
    }
}
```

- `page.addTargetRequests(links)`

  Add urls for crawling.

You can also use annotation way:

```
@TargetUrl("https://github.com/\\w+/\\w+")
@HelpUrl("https://github.com/\\w+")
public class GithubRepo {

    @ExtractBy(value = "//h1[@class='entry-title public']/strong/a/text()", notNull = true)
    private String name;

    @ExtractByUrl("https://github\\.com/(\\w+)/.*")
    private String author;

    @ExtractBy("//div[@id='readme']/tidyText()")
    private String readme;

    public static void main(String[] args) {
        OOSpider.create(Site.me().setSleepTime(1000)
                , new ConsolePageModelPipeline(), GithubRepo.class)
                .addUrl("https://github.com/code4craft").thread(5).run();
    }
}
```

### Docs and samples:

Documents: http://webmagic.io/docs/

The architecture of webmagic (refered to Scrapy)

Javadocs: http://code4craft.github.io/webmagic/docs/en/

There are some samples in `webmagic-samples` package.

### Lisence:

Lisenced under Apache 2.0 lisence

### Contributors:

Thanks these people for commiting source code, reporting bugs or suggesting for new feature:

- ccliangbo
- yuany
- yxssfxwzy
- linkerlin
- d0ngw
- xuchaoo
- supermicah
- SimpleExpress
- aruanruan
- l1z2g9
- zhegexiaohuozi
- ywooer
- yyw258520
- perfecking
- lidongyang
- seveniu
- sebastian1118
- codev777
- fengwuze

### Thanks:

To write webmagic, I refered to the projects below :

- **Scrapy**

  A crawler framework in Python.

  http://scrapy.org/
- **Spiderman**

  Another crawler framework in Java.

  https://gitcafe.com/laiweiwei/Spiderman

### Mail-list:

https://groups.google.com/forum/#!forum/webmagic-java

http://list.qq.com/cgi-bin/qf\_invite?id=023a01f505246785f77c5a5a9aff4e57ab20fcdde871e988

QQ Group: 373225642

- Status
- API
- Training
- Shop
- Blog
- About
- Pricing


- © 2016 GitHub, Inc.
- Terms
- Privacy
- Security
- Contact
- Help

Something went wrong with that request. Please try again.

You signed in with another tab or window. Reload to refresh your session.
You signed out in another tab or window. Reload to refresh your session.
