# Supplementary material for: Construction of a rural tourism information service management system for multi-source heterogeneous data processing
Source: PeerJ Comput Sci. 2023 Jun 9;9:e1334. doi: 10.7717/peerj-cs.1334 (PMC10280680; doi:10.7717/peerj-cs.1334)
Supplement: Supplemental Information 2 [file peerj-cs-09-1334-s002.zip › Code/webmagic-extension/src/test/resources/html/mock-webmagic.html]

20170603

12


- 1
- 2
- 3
- 4

- 20170601
- 20170602
- 20170603
- 20170604
